# Implementation of Mobile Phone Data Collection in the Conduct EPI Comprehensive Review in East and Southern African Countries

**DOI:** 10.29245/2578-3009/2021/S2.1108

**Published:** 2021-04-13

**Authors:** Isah Mohammed Bello, Abubakar Sadiq Umar, Godwin Ubong Akpan, Joseph Okeibunor, Chukwudi Shibeshi, Messeret Eshetu, Chakauya Jethro Magwati, Teshager Fasil, Daniel Fussum, Richard Mihigo, Pascal Mkanda

**Affiliations:** 1 WHO East & Southern Africa Support Team (WHO ESA IST); 2 WHO Regional Office for Africa (WHO AFRO)

## Abstract

Mobile phone data collection tools are increasingly becoming very usable collecting, collating and analysing data in the health sector. In this paper, we documented the experiences with mobile phone data collection, collation and analysis in 5 countries of the East and Southern African, using Open Data Kit (ODK), where questionnaires were designed and coded on an XML form, uploaded and data collected using Android-Based mobile phones, with a web-based system to monitor data in real-time during EPI comprehensive review. The ODK interface supports in real-time monitoring of the flow of data, detection of missing or incomplete data, coordinate location of all locations visited, embedded charts for basic analysis. It also minimized data quality errors at entry level with the use of validation codes and constraint developed into the checklist. These benefits, combined with the improvement that mobile phones offer over paper-based in terms of timeliness, data loss, collation, and real-time data collection, analysis and uploading difficulties, make mobile phone data collection a feasible method of data collection that needs to be further explored in the conduct of all surveys in the organization.

## Introduction

A National Immunization Programme Review also referred to as Expanded Programme on Immunization (EPI) Review, is a comprehensive assessment of the strengths and weaknesses of an immunization programme at national, subnational and service-delivery levels, with the sole aim of providing evidence to guide programme’s strategic directions and prioritizing activities^7^. The review requires a collection of data from tools, through the conduct of administering questionnaires; observations; review of data, documents, and reports; such as tally sheets and immunization registers, monthly reports and electronic databases of health management information system (HMIS) data (e.g. DHIS2) etc. During previous EPI reviewed a standard paper based questionnaires were used to collect information which requires further collation and analysis which is labourous and time consuming^7^. Paper-based system requires data to be entered manually leading to errors in the data, delays in data cleaning and analysis which ultimately does not facilitate availability of information same day and real time^8,12^. A study in Pemba (Zanzibar- Tanzania) raises concerns of time interval between data collection and checking, leading to delay in resolving the data issues. It also noted omission errors and illogical data, as well as inconsistency in enrolment criteria resulting in missing eligible clients which had made the tracking of the proportion of clients missed difficult^12^. Use of paper-based system has been reported to be a time-consuming and difficult when dealing with large data sets^2^. Another challenge of using paper-based approach in the conduct of EPI reviews was the delay between the data collection and the collation and analysis of data to be used for preparing the report, this usually take days before, with missing and incomplete data sets affecting the quality of the feedback used for the report^9^.

To overcome the above-mentioned challenges with paper-based approach, there has been an attempt to use data collection applications on smartphones and tablets in the conduct of EPI Review in Tanzania in the year 2015. This attempt met with challenges, which include, time to translate the tools into mobile application, difficulty in transmitting data due to poor internet services, and timing for training^6^. The use of such technology is not only limited to surveys but has been used in various clinical settings. For instance, studies conducted across different country settings have tried to investigate the use ofcell phones on the patient end in an effort to generate feedback for improved chronic illness care and monitoring^5,12^ and monitoring pregnant women in remote areas in Liberia^12^. Furthermore, innovative technologies such as the personal digital assistants (PDAs) have been used to document vital health information and the health seeking behaviour among 21,000 rural dwellers in southern provinces of Tanzania^11^. Additionally, other studies have investigated the use of cell phones on the linking smartphones to web applications for epidemiology, ecology and community data collection^1^ or used in clinical microscopy for global health applications purposes^3^. In general, in poor resource settings, the Open Data Kit (ODK) is commonly used because it is free, easy to implement in terms of coding, can work both realtime, online and offline (during data collection) without the need for internet connection as well as user-friendly to the interviewer.

In order to improve real time information on surveillance on other health service-related issues, the Regional office of the World Health Organisation has established the Geographical Information (GIS) centre and has directed that the Polio and IVD programs initiate the use of online surveillance tools (active case search and integrated supportive supervisory tools from 2017. As of week, 31, 2020 a total of 83,981 cumulative supportive supervision visits have been conducted in 44 African countries as compared to 62,456 cumulative supportive supervision visits in 2019 (ref). Despite the increase in the availability of real time AFP active surveillance data (same day of visit) that improved program decision, however, there eare no published studies on the use of mobile phones as a data collection tool in the conduct of EPI reviews in the African region.

This paper documents the processes of using mobile phone data collection in the conduct of EPI reviews, to highlight the lessons learnt, opportunities and challenges with a view to addressing the gaps and limitations. This work also explores opportunities towards ensuring a timely, accurate and real-time data to support timely decision-making processes by program managers that will improve programme performance.

## Methods

### Study design

This is a primary data analysis of the EPI Comprehensive Review Checklists, conducted in the 5 countries that implemented the mobile phone data collection method between September 2017 to September 2018 in the sub-region

### Study area

The study was conducted in five countries namely; South Sudan, South Africa, Ethiopia, Mauritius, and Kenya. The countries have an estimated population <1 year and <15-year population of 6,013,192 and 78,100,994 respectively. Currently, there are a total of 5 countries in the sub-region that implemented the use of ODK during the conduct of the review, with over 70 different questionnaires containing over 3,500 records already available on the WHO AFRO server.

### Study population

A total of 5 countries in the sub-region were selected and included in the study based on the criteria of whether a country had used paper based or electronic ODK based questionnaires during the most recent EPI review that was conducted between September 2017 to September 2018.

## Open Data Kit (ODK): The Mobile Phone Data Collection System

**Figure 1:**
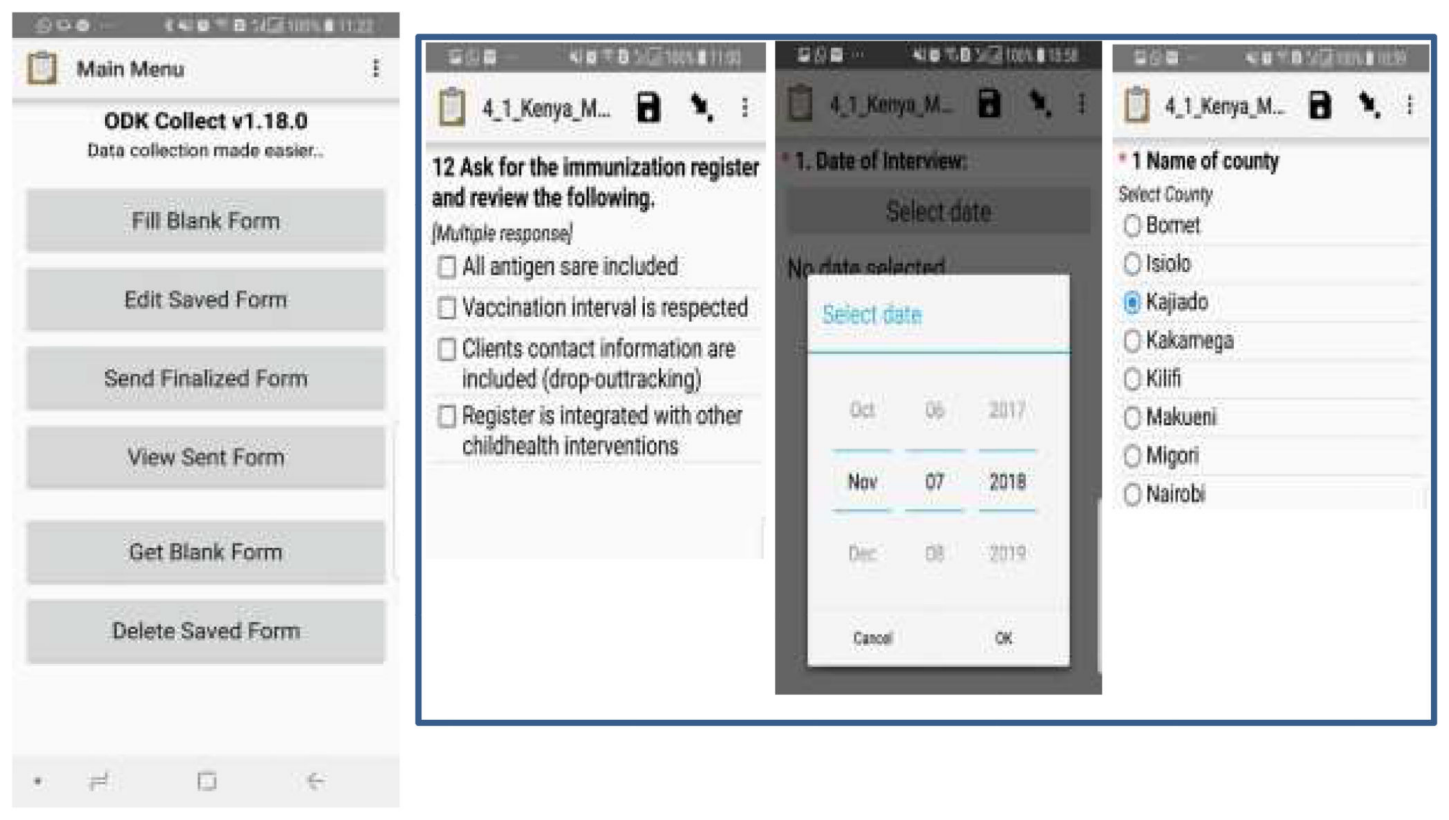
Screen shots of a questionnaire on the mobile phone.

Open Data Kit (ODK) is a set of tools that supports data collection using mobile and hand held devices, with enhanced capability of data submission to an online server, even without an Internet connection or mobile carrier service at the point collection. Data collected from the field with ODK Collect, can be uploaded and managed using ODK Aggregate. ODK Aggregate is the intermediary platform used as a server storage, which accepts data and relay it to external applications. The ODK Aggregate enable the user to download a specified dataset in different file formats, such as xls, html, json, csv etc. It allows you to use Google’s App Engine, that is hosting the platform managing the data collected remotely in real-time. It was created by developers at the University of Washington’s Computer Science and Engineering department and members of Change, Open Data Kit is an open-source project available to all. The user has the option to save the data as Complete or Incomplete. If the questionnaire is completed and saved as COMPLETE, the data moves to the SEND FINALISED FORM, from which it is automatically uploaded to the server. If there is no mobile network coverage, completed questionnaires are stored securely in the phone until a signal is found and the data is automatically uploaded. ODK can incorporate multiple choice, free text, numeric, date, time and other question types (see Figure 1).

In addition, ODK can also accommodate data entry constraints, skip logic as well as enforced validation in the field to reduce errors at the entry level. The data are uploaded using low-cost general packet radio service (GPRS). The ODK platform has a web-based interface that was developed to facilitate the review of data and exporting of results in standard file formats such as comma separated values (CSV), Microsoft Excel, Microsoft Access etc. The data teams communicate with all the teams in the field directly, either through a call to the mobile phone or through SMS messaging. A WhatsApp group was equally created to ease communication and provide support to the team in the field. A dashboard was created on the ODK web-based interface on basic indicators from the survey that are automated in real-time, which provided the programme officers the ability to monitor status of data submission, while the survey is going on (these includes, locations visited based on coordinates taken, number of records available on the server etc.).

All the data were sent to the WHO AFRO server which was secured by firewalls to prevent unauthorized access and denial of service attacks. Access to the ODK web-interface is protected by passwords. For the purpose of these reviews, access to the data was granted to all programme officers using the read-only right, and the data managers have the administrative right that allows for edition (where necessary).

## Data collection and Training

Different teams (between 6 to 8) were formed comprising people with different specialties (epidemiologists, communications specialists, data managers, logisticians etc.), which were assigned to different provinces.

The data were collected by the team in all the selected categories agreed (national, regional, districts and health facilities). The Focal person for each of the thematic areas (surveillance, EPI, cold chain, data management, communications) in each level was interviewed. All interviews were conducted face-to-face, in the offices and locations of the focal persons using standard questionnaires.

Android-based mobile phones/tablets were provided for the data collection process and most of the team members had previous experience in the use of Android-based smartphones. Training on the use and configuration of the open data kit (ODK) was conducted before exposing team members to the data collection protocol over a two-day period. The training consisted of a general orientation on using the phone and its data collection application (ODK), downloading and accessing the questionnaires on the phone, data dictionary for each question on the questionnaire and standard care of the device. The training package involved troubleshooting mobile devices, as well as steps for configuring the new or existing devices, to ensure that all data collectors are able to deal with basic technical issues that may arise with the use of the mobile phones in the field. Survey questions were pilot tested during the training prior to implementation to ensure that all the skip logic, constraints, and validation applied are working and to ensure that the questions were understandable to respondents. The data tools (questionnaire) reviewed based on comments from data collection team members, and programme officers from government and partner agencies. The method for data collection was interviewer administered questionnaire. Standard protocols for administering the questionnaires were developed which provided a uniform guidelines on how go through the questionnaires.

The data collection phase was conducted within five working days in all the countries except in South Sudan (where it varies due to insecurity and availability of logistics). Data are shared directly from the field real-time (where there is network) and data flow is being monitored by the data managers, identifying missing information (if any) and providing daily feedback to the teams and the programme managers on the status of data on the server, and inconsistencies and missing information are communicated, rectified, and cleaned in a timely manner. No device failure or application problem was recorded throughout the course of the survey, except for few delays in getting geo-coordinates in some locations, which were addressed immediately.

## Results

The use of the mobile based android device in the conduct of the EPI reviews had shown a significant improvement in the timeliness and completeness of the data, with real-time submission of the data from the field as seen in the table below;

We noticed from the result that the timeliness and completeness of the data is greater than 95% in all the countries that implemented, proportion of errors found on the data after collation has also improved with an average of 0.01 to 0.003, while number of days taken to collate the data for analysis is 1 day (data submission was real-time). Standard charts and maps were automatically created on the web-based platform and saved to the DASHBOARD, which allowed the review lead team to visualize real-time outputs of the location of visits, number of records per location, geo-codes of areas where the questionnaire were administered on a daily basis. Outputs can be generated when the person wants to analyze the data using external tools (e.g., EXCEL) as often as the user prefers.

**Figure 3:**
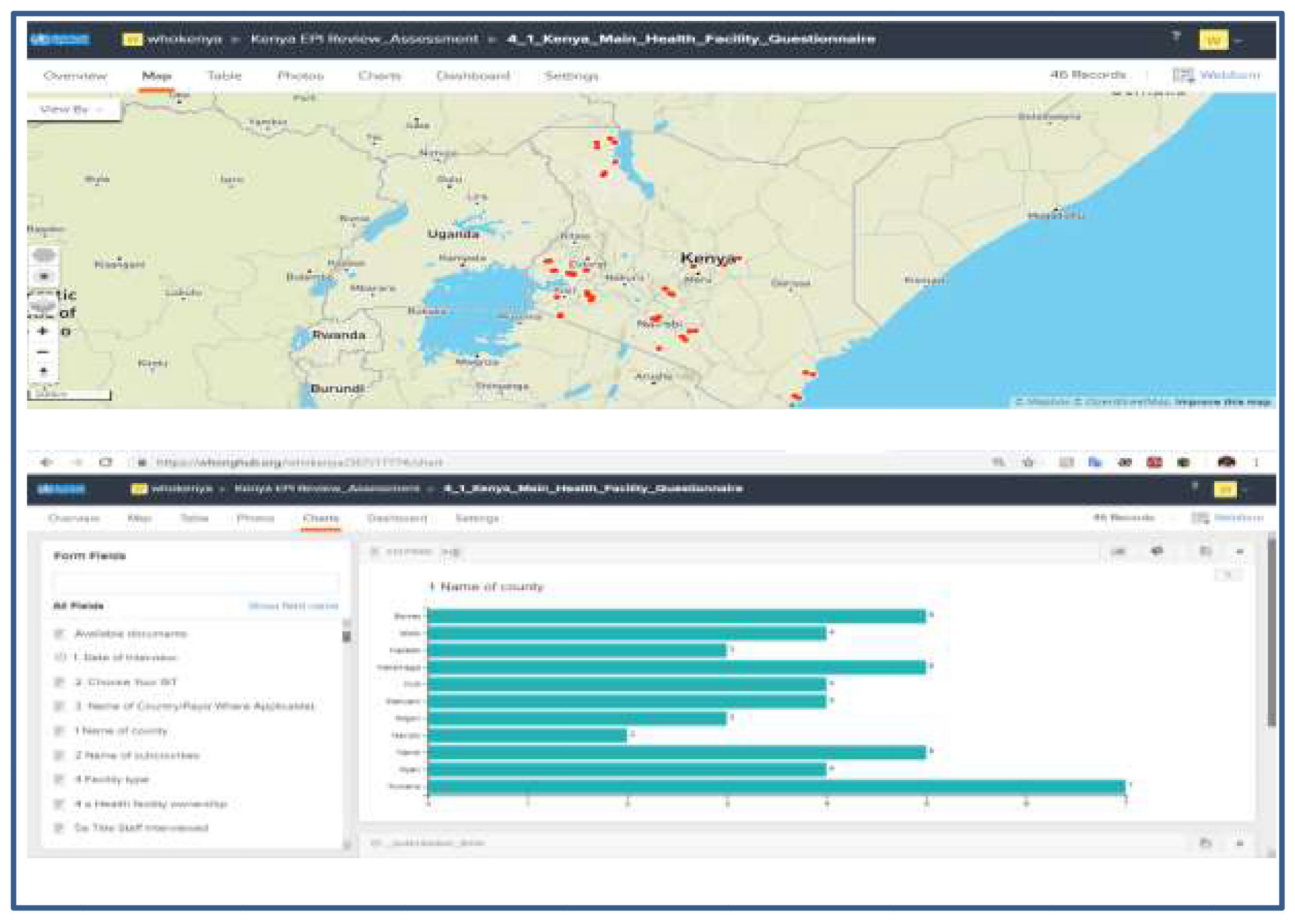
Sample output of the interface showing locations visited and number of records per site visited in charts

Alternatively, built-in graphs and reports on the ODK web -based interface permit real-time visualization of the data, which includes maps, charts (Figure 3) and save it on to the dashboard for real-time data visualization.

The use of the mobile phone and the web-interface using ODK, allowed the External Team Leads the opportunity to monitor work rate, geographical distribution of the sites visited in each location (Figure 3). The automatic send option was enabled on all the mobile phones, which allows completed questionnaire to be shared automatically whenever there is a network. Daily meetings between the data manager and the national team, chaired by the external team lead were held to know the status of data available in the server and share any experiences/difficulties reported from the field for correction. A daily summary of data available on the server was shared with all teams for follow up and feedback.

**Figure 4:**
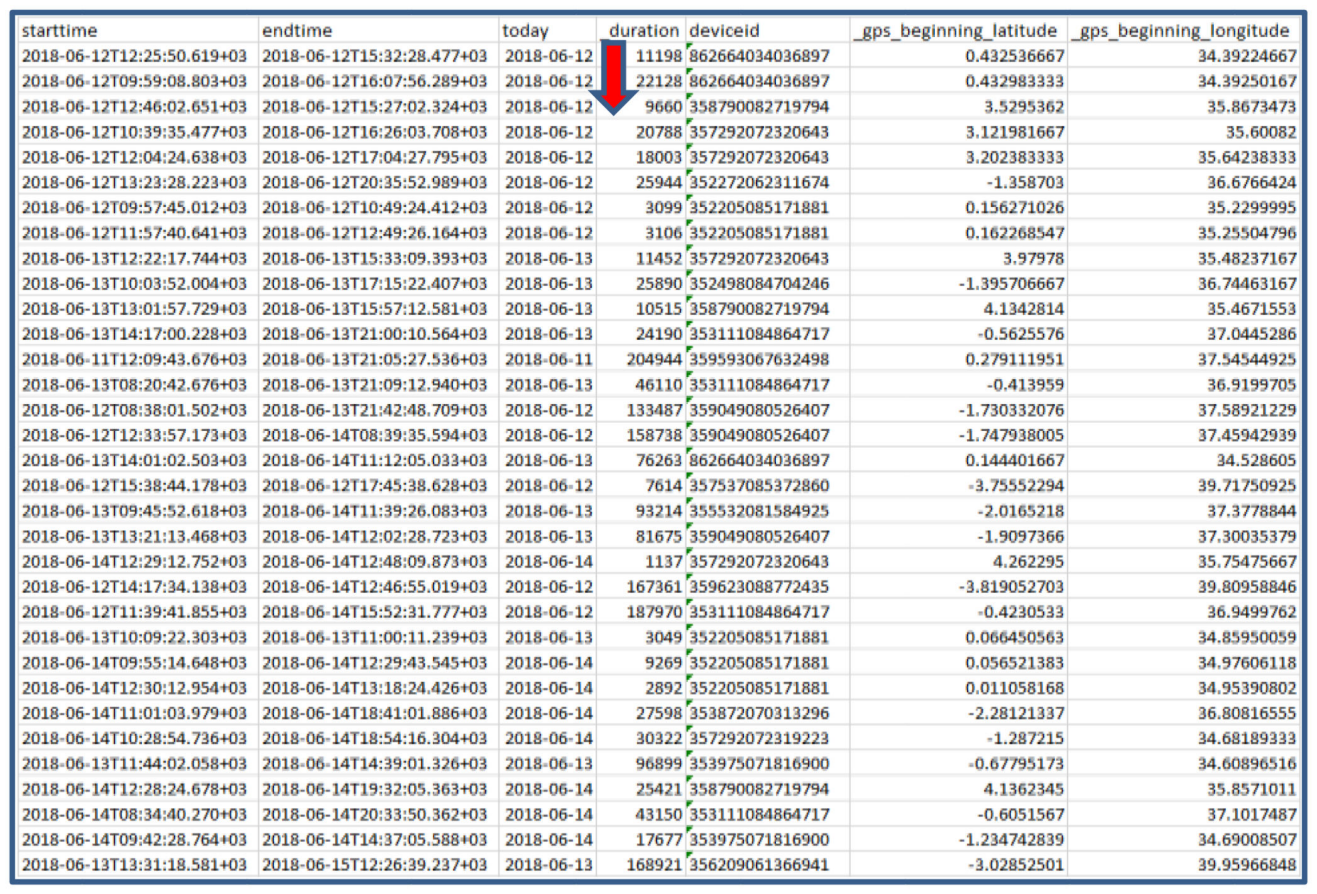
Extract of Exported output showing the start time, end time and duration of the questionnaire

One of the major advantages of the mobile phone data collection method was in the real-time detection of suspicious data entry and data falsification. The exported data report provides formatted information files, the time a specific questionnaire was administered, the device_id, latitude, longitude, duration (the time stamp analysis) etc, which you can use to calculate the time taken to finish administering the questionnaire (Fig. 4).

There was a scenario where it was discovered that in a health facility conducting routine immunization session live, it took the surveyor less than 5 minutes to administer the checklist [This was thought to be a short time given our expectations about the amount of time it would reasonably take to observe a session, conduct client exit interview etc.]. The team lead immediately alerted to validate the visit and call the attention of the surveyor to understand the situation, it was later found out that the surveyor finished the activity first and later came and filled the checklist at once. This information was used to further sensitize the other members on the best approach to handle the mobile.

During the survey, some of the challenges identified includes the issue of power as some of the mobile phones could not last the whole day and went off during data collection. Additionally, the lack of network and internet connection in some locations could not allow some of the surveyors to share the data from the field as they had to wait till when they reach an area with good network which resulted in delays to submit completed questionnaires.

## Discussion

In general, our findings show that mobile phonebased data collection (using ODK) has greatly improved real-time data collection during the review. This is a great improvement when compared to previous reviews, where data collation took 3 days in the 2016 EPI review for Mozambique^15^, some of the data from health facilities got lost during collation and affected the completeness of the data^13^. The reason for the remarkable improvement compared to the paper based approach is due to the fact that the ODK application platform (both mobile and webbased interface) enhanced real-time supervision and monitoring the flow of data, which markedly improved our ability to detect data fabrication and suspicious data entries^4^. Furthermore, the number of errors during data entry was low because of the inherent data entry constraints, skip logic and enforced validation on the ODK application unlike the paper based approach that has been reported to be prone to errors of omission^4^.

Nevertherless, it is pertinent to note that the application does not negate data fabrication, which could still go undetected. For example, a data collector could key in wrong figures for population and as long as they are numbers (integers) it will not be detected as an error. This disadvantage might be reduced through verification because the application has GPS capabilities which allowed supervisors to know the locations visited by the surveyor as geo-codes of each location is required before and after the completion of the data entry. Hence, any suspicious entry can be revalidated since the coordinates for each location was recorded and therefore, traceable for verification. The web-based interface has other advantages such as automated graphs, charts deployed on the ‘DASHBOARD’ and real-time information allowed the team lead and the programme manager’s opportunity to focus their time on other aspects of the review and solving logistical difficulties in the field.

In the conduct of these reviews, in all the countries except Mauritius, we had used the existing mobile phones that were purchased for other programs to enter and upload data at the point of collection. This allows us to implement the process with little or no direct costs of phones. While it may be difficult to compare the costs incurred with using a paper-based approach in this study, it is most likely that the cost of using the mobile phones compares favourably to a paper-based approach. The issue of the cost-effectiveness of the system needs to be explored in future studies that compares the paper-based approach and the phone-based data collection approach.

The automatic sending of data from the mobile phone to the server significantly increases the timely submission of data. In our study, there was no data loss experienced and data that were not sent automatically (due to lack of internet services in the field) were later sent in areas with internet connectivity. This further underscored the benefits of using innovative technology to collect data. However, this finding was inconsistent with other studies where the issue of loss of data due to technical problems, damage, theft or loss of phones was reported^10^. In our study, the loss of data was partially minimized because of the little time lapse between data collection and data upload.

The issue of power to charge the phones in the field that was reported as a challenge could be mitigated by having a power bank, which could be used in the field, while sending of data could be delayed until the team reached a location with good internet access. One of the limitations of the findings of this study is that the design did not consider comparison of the time spent between using paper-based and using the mobile phone data collection and the cost implication of each approach which calls for further studies using a control group to compare the two systems. However, the opportunity of automatic sending of data where network/internet exist and the real-time access significantly improves the timeliness of data, alongside the skip patterns and constraints adapted in the checklist while collecting data provided the ability to validate the data during collection and ensures quality data reaches the server.

## Conclusion

The experience gathered during the EPI review in these countries clearly shows that mobile data collection enabled with the real-time monitoring of data flow improves the timeliness and quality of the data, thereby making it an interesting approach as compared the previous paper-based approach. It can support processes that require collecting data from the field using superb validation and can be adopted for all relevant program reviews of different types and sizes. The gains associated with mobile technology, plus the advantage in relation to coordinates location capture that mobile phone offers, ease of use and constraints at entry level makes it a preferred solution of data collection that should be explored.

## Figures and Tables

**Figure 2:**
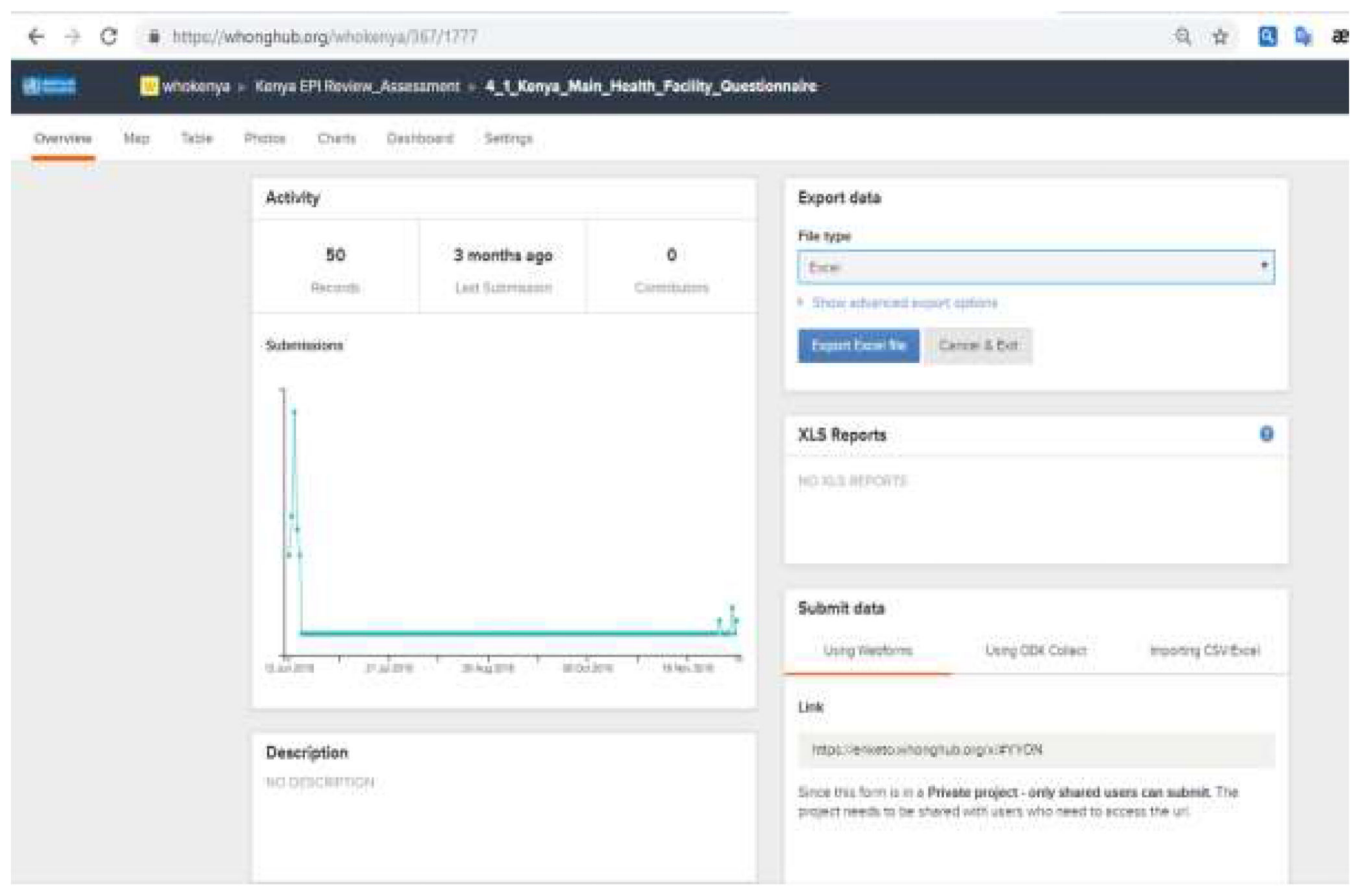
Sample output of exporting the data to an external application (e.g. EXCEL) to be analysed

**Table 1:** Summary of Checklist Questionnaire by Country

Countries	No of Provinces Visited	No of Districts Visited	No of HFs Visited	National Level Questionnaire	Province Level Questionnaire	District Level Questionnaire	HF Level Questionnaire	Total Number of Checklists Expected
Ethiopia	7	20	80	5	40	40	240	325
Kenya	7	22	88	5	44	44	264	357
Mauritius	6	6	12	4	18	12	36	70
South Sudan	8	20	80	5	40	40	240	325
South Africa	8	20	80	5	40	40	240	325

* National Level (Surveillance, EPI, Laboratory, NITAG Chair and Partners)

* Province Level (Surveillance, EPI and Partners)

* District Level (Surveillance and EPI)

* HF Level (Surveillance, EPI and Exit interview)

**Table 2:** Summary of percentage of timeliness, completeness and errors by country

Countries	No of Province Visited	Total Number of Checklists Expected	Timeliness	Completeness	Number of Checklist with errors	Checklist with Errors (%)	No of Days taken to collate data
Ethiopia	10	325	95	97	5	0.02	2
Kenya	11	357	98	100	9	0.03	1
Mauritius	6	70	96	100	1	0.01	1
South Sudan	10	325	95	97	4	0.01	2
South Africa	10	325	98	99	5	0.02	2
